# Supplementary material for: A spectacular new species of Hyloscirtus (Anura: Hylidae) from the Cordillera de Los Llanganates in the eastern Andes of Ecuador
Source: PeerJ. 2022 Sep 29;10:e14066. doi: 10.7717/peerj.14066 (PMC9527025; doi:10.7717/peerj.14066)
Supplement: Supplemental Information 1 — Results of phylogenetic analisys are shown, supporting the new species within Hyloscirtus larinopygion species group [file peerj-10-14066-s001.docx]

**Phylogenetic relationships.** Our phylogenetic analysis (Fig. 1) shows that the new species is sister to a clade consisting of *Hyloscirtus criptico* Coloma et al. (2012)*, H. larinopygion* (Duellman, 1973)*, H. lindae* (Duellman & Altig,1978)*, H. pach*a (Duellman & Hillis, 1990), *H. pantostictus* (Duellman & Berger*,* 1982), *H. princecharlesi* Coloma et al. (2012)*, H. psarolaimus* (Duellmn & Hillis, 1990), *H. ptychodactylus* (Duellman & Hillis, 1990)*, H. staufferroum* (Duellman & Coloma, 1993) and , *H. tigrinus* Mueses-Cisneros & Anganoy-Criollo (2008)*.* Genetic distances (mitochondrial 16S percent differences calculated from uncorrected p values between the new species and the most closely related *Hyloscirtus* are given in Table 1; genetic distances between the new species and its closest relative were 2.2-2.9% to *H. tigrinus* and 2.6-2.8% to *H. ptychodactylus*.

**Figure 1**: Evolutionary relationships of species in the *Hyloscirtus larinopygion* group, based on the mitochondrial gene 16S under ML criterion. Clade support (bootstrap %) are in blue. The new species is in red.


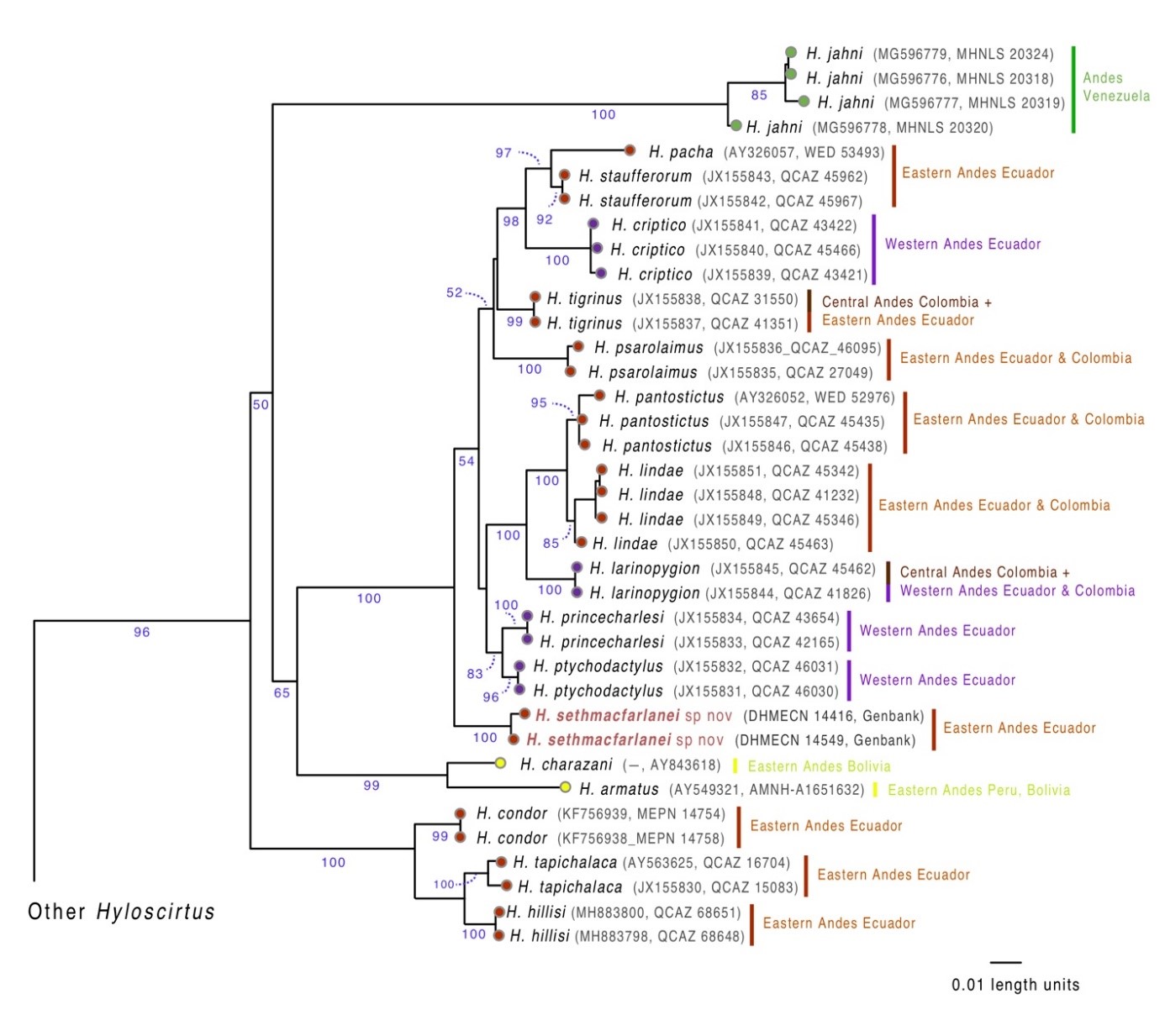


**Table 1.** Genetic distances (mitochondrial 16S) between *Hyloscirtus sethmacfarlanei* *sp. nov*. and its most closely related congeners. Values are presented as percent distances calculated from uncorrected *p* values.

|  | ***H. condor***  **(n = 2)** | ***H. hillisi***  **(n = 5)** | ***H. pacha***  **(n = 1)** | ***H. larinopygion* (n = 2)** | ***H. lindae* (n = 4)** | ***H. pantostictus* (n = 3)** | ***H. tapichalaca***  **(n = 2)** | ***H. ptychodactylus* (n = 2)** | ***H. princecharlesi* (n = 2)** | ***H. psarolaimus* (n = 2)** | ***H. tigrinus* (n = 2)** | ***H. staufferorum* (n = 2)** | ***H. sethmacfarlanei* sp nov (n = 2)** |
| --- | --- | --- | --- | --- | --- | --- | --- | --- | --- | --- | --- | --- | --- |
| ***H. condor*** | 0.0 |  |  |  |  |  |  |  |  |  |  |  |  |
| ***H. hillisi*** | 3.8–4.1 | 0.0–0.1 |  |  |  |  |  |  |  |  |  |  |  |
| ***H. pacha*** | 10.2–10.5 | 11.3–11.4 | 0.0 |  |  |  |  |  |  |  |  |  |  |
| ***H. larinopygion*** | 9.6–9.8 | 10.2–10.4 | 4.6 | 0.0 |  |  |  |  |  |  |  |  |  |
| ***H. lindae*** | 10.1–10.8 | 11.0–11.7 | 4.6–5.3 | 2.6–3.1 | 0.0–0.5 |  |  |  |  |  |  |  |  |
| ***H. pantostictus*** | 10.4–10.8 | 11.1–11.5 | 4.7–4.8 | 2.7–2.8 | 6.0–14.0 | 0.0–0.1 |  |  |  |  |  |  |  |
| ***H. tapichalaca*** | 3.7–4.0 | 2.7–3.1 | 11.0–11.1 | 10.1–10.4 | 10.9–11.4 | 11.1–11.4 | 0.6 |  |  |  |  |  |  |
| ***H. ptychodactylus*** | 9.5–9.6 | 10.2–10.5 | 3.8 | 2.9–3.0 | 2.9–3.6 | 3.1–3.2 | 10.1–10.2 | 0.0 |  |  |  |  |  |
| ***H. princecharlesi*** | 9.7–9.9 | 10.8–11.0 | 4.3 | 3.2 | 3.3–3.6 | 3.4–3.6 | 10.6–10.7 | 1.3 | 0.0 |  |  |  |  |
| ***H. psarolaimus*** | 10.8–11.1 | 11.3–11.7 | 4.2–4.5 | 4.8–5.0 | 4.5–5.4 | 4.8–5.2 | 11.2–11.4 | 3.6–3.8 | 3.8–4.1 | 0.3 |  |  |  |
| ***H. tigrinus*** | 9.8–10.1 | 10.5–10.9 | 3.1–3.2 | 3.9–4.0 | 3.9–4.6 | 4.0–4.2 | 10.5–10.8 | 2.6 | 2.8–2.9 | 3.0–3.3 | 0.0 |  |  |
| ***H. staufferorum*** | 9.6–10.1 | 10.6–11.1 | 1.8 | 4.2–4.4 | 4.0–4.7 | 4.1–4.3 | 10.6–10.8 | 2.8–2.9 | 3.3–3.4 | 3.4–3.7 | 2.7–2.8 | 0.0 |  |
| ***H. sethmacfarlanei sp nov*** | 9.3–9.5 | 9.2–9.6 | 3.7–3.9 | 2.9–3.5 | 3.7–4.6 | 3.5–3.7 | 9.4–9.9 | 2.6–2.8 | 3.1–3.3 | 3.1–3.3 | 2.2–2.9 | 3.5–4.0 | 0.4 |
